# P12 Investigating the dynamic interaction between *Salmonella* and intestinal microbiota in health and disease

**DOI:** 10.1093/jacamr/dlag102.018

**Published:** 2026-06-26

**Authors:** Asmaa T Alenazi, Anjam Khan, Christopher Nile, Chien-Yi Chang

**Affiliations:** School of Dental Sciences, Faculty of Medical Sciences, Newcastle University, NE2 4BW, UK; Department of Biology, College of Science, Northern Border University, Saudi Arabia; Bioscience Institute, Faculty of Medical Sciences, Newcastle University, Newcastle, NE2 4HH, UK; School of Dental Sciences, Faculty of Medical Sciences, Newcastle University, NE2 4BW, UK; School of Dental Sciences, Faculty of Medical Sciences, Newcastle University, NE2 4BW, UK; Bioscience Institute, Faculty of Medical Sciences, Newcastle University, Newcastle, NE2 4HH, UK

## Abstract

**Background:**

Antimicrobial resistance (AMR) is an increasing global concern. The spread of MDR *Salmonella enterica* strains has limited the effectiveness of many commonly used antibiotics for treating gut infections, creating a clear need for alternative ways to reduce pathogen colonization and control infections. The human gastrointestinal tract is inhabited by a complex microbial ecosystem, which plays a key role in maintaining host health and mediating resistance to diseases. One potential strategy is the use of probiotics, which can provide colonization resistance by competing with pathogens and producing antimicrobial factors within the gut environment. *Escherichia coli* Nissle 1917 (EcN) is a widely used probiotic with established immunomodulatory properties and clinical relevance in inflammatory bowel diseases. EcN expresses a K5 polysaccharide capsule, a feature typically associated with extraintestinal *E. coli* strains; however, its functional role in bacterial fitness and interspecies competition within the gut environment remains incompletely understood.

**Objectives:**

To investigate the role of the K5 capsule in EcN physiology and competitive behaviour against clinically relevant pathogens, including the non-typhoidal *Salmonella enterica* serovar Typhimurium 4/74 strain and the MDR, invasive D23580 strain.

**Methods:**

Targeted gene deletions of capsule export genes (*kpsE*, *kpsM*, and *kpsT*) in EcN were generated. Phenotypic characterization of wild-type and mutant strains was performed using nigrosin-negative staining to confirm capsule presence, alongside growth curve analyses in both rich (LB) and M9 minimal media to assess bacterial fitness under different nutrient conditions. To evaluate the role of the capsule of EcN in survival competition with *Salmonella* pathogens, both contact-dependent co-culture assays and contact-independent conditioned media (CM) assays were employed.

**Results:**

The data showed that loss of the K5 capsule in the Cell Free Supernatant (CFS) reduced the EcN fitness and allowed *Salmonella* to increase growth.

**Conclusions:**

This work provides a systematic investigation of the EcN K5 capsule to elucidate its role in bacterial fitness and colonization resistance in competing with pathogens. These findings contribute to a deeper mechanistic understanding of how probiotic-associated surface structures influence interbacterial interactions and pathogen exclusion within the gut ecosystem.

